# No Difference in the Pathogenic Microorganisms Among Different Types of Anorectal Abscesses: A Retrospective Study

**DOI:** 10.7759/cureus.56504

**Published:** 2024-03-19

**Authors:** Jun Du, Yangyang Miao, Shuguang Zhen, Qingyun You, Jing Guan, Zongqi He

**Affiliations:** 1 Department of Anorectal Surgery, Suzhou Traditional Chinese Medicine (TCM) Hospital Affiliated to Nanjing University of Chinese Medicine, Suzhou, CHN; 2 Department of Clinical Laboratory, Suzhou Traditional Chinese Medicine (TCM) Hospital Affiliated to Nanjing University of Chinese Medicine, Suzhou, CHN; 3 Department of Medicine, The First Clinical Medical College, Nanjing University of Chinese Medicine, Nanjing, CHN; 4 Department of Anorectal Surgery, Suzhou Traditional Chinese Medicine Hospital, Suzhou, CHN

**Keywords:** clinical infectious disease, retrospective analysis, microorganisms, pus culture, anorectal abscesses

## Abstract

Objective: There is limited data on the pathogenic microorganisms associated with anorectal abscesses. The purpose of this study was to retrospectively analyze the types and quantities of pathogenic microorganisms in the pus cultures of patients with anorectal abscesses and to explore the correlation between pathogenic microorganisms and types of anorectal abscesses.

Methods: A retrospective analysis was conducted on the microbiological data of 517 inpatient surgical patients with anorectal abscesses treated at a single center from January 2017 to December 2021. Chi-square tests were used to analyze whether there were differences in the types and quantities of pathogenic microorganisms among different types of anorectal abscesses.

Results: Among the 517 patients, the mean age was 38.5 years, with an average duration of illness of 7.4 days. Of these, 440 (85.1%) were male and 77 (14.9%) were female. The types of anorectal abscesses included perianal abscesses (54 cases, 10.4%), intersphincteric abscesses (253 cases, 48.9%), ischiorectal abscesses (129 cases, 25.0%), deep posterior anal space (DPAS) abscesses (26 cases, 5.0%), supra-levator abscesses (10 cases, 1.9%), and horseshoe abscesses (45 cases, 8.7%). A total of 23 different microorganisms were cultured from the 517 pus specimens. The most common microorganism was Escherichia coli (323 cases, 62.5%), followed by Klebsiella pneumoniae (77 cases, 14.9%), Bacteroides fragilis (nine cases, 1.7%), Pseudomonas aeruginosa (eight cases, 1.5%), and Staphylococcus aureus (seven cases, 1.4%). Additionally, no microorganisms were cultured from 58 (11.2%) pus specimens. Nine patients (1.7%) were admitted with concomitant necrotizing fasciitis. Among the nine cases of concurrent necrotizing fasciitis, E. coli, K. pneumoniae, and S. aureus were cultured in six (66.7%), two (22.2%), and one (11.1%) case, respectively. Chi-square tests revealed no significant differences in the types and quantities of pathogenic microorganisms among different types of anorectal abscesses (P > 0.05).

Conclusion: This study provides a large sample of pus culture microbiological data from patients with anorectal abscesses. Regardless of whether it is a simple anorectal abscess or concurrent necrotizing fasciitis, E. coli was the most common microorganism cultured from the pus of patients with anorectal abscesses. Other common microorganisms include K. pneumoniae, B. fragilis, and S. aureus. These results provide evidence for the precise antibiotic treatment of anorectal abscesses. Additionally, there were no differences in the types and quantities of pathogenic microorganisms among different types of anorectal abscesses.

## Introduction

Anorectal abscess is one of the most common diseases in the field of colorectal surgery, with an incidence of 8.6-20 cases per 100,000 people, affecting males more severely than females, with an incidence of 2.4-3:1, and a mean age of 40 years [1]. The onset of the disease is acute, with rapid progression and greater pain. Based on the different interstitial spaces involved in the abscess, anorectal abscesses are generally classified as perianal abscess, intrasphincteric abscess, ischiorectal abscess, deep posterior anal space (DPAS) abscess, supra-levator abscess, and Horseshoe abscess [2]. The etiologic factors of anorectal abscesses are multiple, and their primary etiology is believed to be caused by infection of the anal glands. Different species of pathogens cause anorectal infections, including Enterobacter, Enterococcus, anaerobes, Streptococcus, and Staphylococcus [1,3].

Prompt incision and drainage remain the mainstay of treatment for anorectal abscesses [2]. Current guidelines recommend adjunctive antibiotic therapy for anorectal abscesses in patients with comorbidities of cellulitis, diabetes, immunosuppression, or signs of sepsis [2]. Anorectal abscesses are prone to recurrence or secondary anal fistula formation after incision and drainage alone, and our previous study found the incidence of secondary anal fistula after incision and drainage of anorectal abscess to be 56.15% [4]. Literature reports that the use of antibiotics after incision and drainage of anorectal abscesses reduces the rate of fistula formation. Those who used prophylactic antibiotics had a significantly lower fistula formation rate than those who did not use any medication. Postoperative use of antibiotics remains protective against fistula formation [5,6]. However, there is still a lack of data analysis related to the microbiology of anorectal abscesses, and with the evolution of bacterial virulence, the emergence of drug resistance, increased use of immunosuppressive drugs, and increased incidence of inflammatory bowel disease, antimicrobial therapy against perianal abscesses need to be approached with greater caution [1].

Based on these perceptions, we asked the question: do pathogenic microbial species and numbers differ between different types of anorectal abscesses? In this study, we retrospectively analyzed the data of 517 patients with anorectal abscesses treated at a single center from January 2017 to December 2021, counted the microbiological characteristics of patients with anorectal abscesses and the types of abscesses, and analyzed whether the species and number of pathogenic microorganisms differed between different types of anorectal abscesses. This study provides a larger sample of pus culture microorganisms in patients with anorectal abscesses, which is useful for better guiding the precise use of antibiotics in patients with anorectal abscesses.

## Materials and methods

Patient selection

This was a retrospective study of 517 patients who underwent incision and drainage of anorectal abscesses between January 2017 and December 2021 in the Department of Anorectal Surgery, Suzhou Hospital of Traditional Chinese Medicine. The inclusion criteria for patients are age over 17 years, hospitalized patients; patients who underwent incision and drainage surgery for anorectal abscess. The exclusion criteria are ages under 17 years, patients who did not undergo standard pus collection and microbial culture, and patients who underwent abscess drainage in the outpatient or emergency department. Different surgeons performed the operations, and pus was obtained from the abscesses for microbiologic culture. For ruptured abscesses, the secretions from the deeper part of the lesion were generally taken by sterile cotton swabs and placed in sterile test tubes for examination. For unresolved abscesses, after strict skin disinfection, pus is extracted with a sterile syringe and sent for examination. The pus is inoculated onto blood agar plates and MacConkey agar plates, with a four-quadrant streak for 24 hours. There are two sets of culture plates, one set for aerobic bacteria and one for anaerobic bacteria. Specimens were sent for examination within 30 minutes after collection, cultured for 24 hours, and routinely tested for anaerobic bacteria. All pus specimens were tested for drug susceptibility using a Mérieux fully automated microbiology identification and drug sensitivity analysis system (VITEK® 2 Compact, France). The operational parameters of the VITEK® 2 Compact system include automatic identification of up to 400 species of bacteria; rapid identification speed, with Gram-negative bacteria taking two to 10 hours and Gram-positive bacteria two to eight hours; antimicrobial susceptibility testing covering both major categories of bacteria, Gram-negative and Gram-positive. The system has a working capacity of 30 test cards. The medical records of this study were reviewed, and the research team recorded data. The Suzhou Hospital of Traditional Chinese Medicine Ethics Committee approved this retrospective study (Approval #: 2022 Ethical Person Approval (053)).

Data collection

All patients underwent incision and drainage under general or regional anesthesia in the operating room. The data collected by the research team included the patient's age, sex, duration of disease, comorbidities (diabetes mellitus, hematologic disorders, inflammatory bowel disease, and severe infections such as necrotizing fasciitis), type of anorectal abscess, and microbiological cultures of the pus.

Statistical methods

Quantitative variables were presented using mean and standard deviation (SD). Categorical variables were described as frequencies (percentages) and compared using Fisher's exact test. Data were statistically analyzed and graphed using GraphPad Prism Version 9.3.1, and differences were considered statistically significant at P < 0.05.

## Results

General data analysis of patients with anorectal abscesses

As shown in Table 1, of the 517 patients, 440 (85.1%) were male, and 77 (14.9%) were female, with a male-to-female ratio of 5.7:1. The mean age was 38.5±12.4 years, and the mean duration of disease was 7.4±10.0 days. In terms of distribution of anorectal abscess types, in descending order, there were 253 (48.9%) cases of intrasphincteric abscess, 129 (25.0%) cases of ischiorectal abscess, 54 (10.4%) cases of perianal abscess, 45 (8.7%) cases of Horseshoe abscess, DPAS abscess 26 (5.0%), and Supra-levator abscess 10 (1.9%). At the time of admission, the patients were known to have leukemia in three (0.6%) cases, diabetes in three (0.6%) cases, and inflammatory bowel disease in two (0.4%) cases.

**Table 1:** General information about the anorectal abscesses patients (n=517) Quantitative variables (Age and Disease duration) were presented using Mean±SD. Categorical variables (sex and type of anorectal abscess) were described as n (%). Abbreviations: SD, standard deviation.

Variable	Values
Sex, n (%)	
Male	440（85.1）
Female	77（14.9）
Age (years), Mean±SD	38.5±12.4
Disease duration (days), Mean±SD	7.4±10.0
Type of anorectal abscess, n (%)	
Perianal abscess	54（10.4）
Intrasphincteric abscess	253（48.9）
Ischiorectal abscess	129（25.0）
Deep posterior anal space abscess	26（5.0）
Supra-levator abscess	10（1.9）
Horseshoe abscess	45（8.7）

Culture results of microorganisms in pus

Tables 2, 3 show that 23 microorganisms were cultured from 517 pus specimens. The most common microorganism was Escherichia coli in 323 cases (62.5%), followed by K. pneumoniae in 77 cases (14.9%), Bacteroides fragilis in nine cases (1.7%), Proteus mirabilis in eight cases (1.5%) and Staphylococcus aureus in seven cases (1.4%). In addition, no microorganisms were cultured from pus specimens in 58 cases (11.2%).

**Table 2:** Microbiological culture results (n=517) The frequency of microorganisms was presented using n (%).

Microorganisms		Frequency, n (%)
Enterobacteriaceae		
	Escherichia coli	323 (62.5)
	Klebsiella pneumoniae	77 (14.9)
	Morganella morganii	2 (0.4)
	Enterobacter spp.	7 (1.4)
	Citrobacter	7 (1.4)
	Proteus	9 (1.7)
	Edwardsiella Ewing and McWhorter	1 (0.2)
Staphylococcus spp.		
	Staphylococcus aureus	7 (1.4)
Streptococcus spp./ Enterococcus spp.		
	Streptococcus spp.	6 (1.2)
	Enterococcus spp.	4 (0,8)
Anaerobes		
	Gram positive	1 (0.2)
	Gram negative	11 (2.1)
Other gram negative bacilli		
	Pseudomonas aeruginosa	1 (0.2)
Cultured two species of bacteria		3 (0.6)
No pathogenic bacteria were cultured		58 (11.2)

Regarding microbial species, Enterobacteriaceae were the most abundant microorganisms, with 426 (82.4%) cases. Escherichiaceae, Klebsiella, Citrobacter, Morganella, Enterobacter, Proteus, and Edwardsiella belong to Enterobacteriaceae. The second place among the microorganisms cultured in the culture was anaerobic bacteria, with 12 cases (2.3%). Starting from the third place in order were Streptococcus spp./Enterococcus spp. 10 cases (2.0%), Staphylococcus spp. seven cases (1.4%) and Pseudomonas aeruginosa one case (0.2%). In three (0.6%) cases, pus culture results showed the presence of two microorganisms, namely P. mirabilis + Enterococcus faecalis, E. coli + K. pneumoniae, and E. coli + P. mirabilis, all belonging to the family Enterobacteriaceae.

**Table 3:** Species and numbers of microorganisms cultured in different types of anorectal abscesses (n=517) The frequency of microorganisms was presented using n (%). Abbreviations: G-：gram negative, NA: No pathogenic bacteria were cultured.

Type of anorectal abscess	Enterobacteriaceae	Staphylococcus spp.	Streptococcus spp.	Anaerobes	Other G^-^ bacilli	Cultured 2 species of bacteria	NA	Total
Perianal abscess	43 (8.3)	2 (0.4)	2 (0.4)	2 (0.4)	0	0	5 (1.0)	54 (10.4)
Intrasphincteric abscess	209 (40.4)	1 (0.2)	6 (1.2)	6 (1.2)	1 (0.2)	3 (0.6)	27 (5.2)	253 (48.9)
Ischiorectal abscess	99 (19.1)	4 (0.8)	1 (0.2)	4 (0.8)	0	0	21 (4.1)	129 (25.0)
Deep posterior anal space abscess	24 (4.6)	0	0	0	0	0	2 (0.4)	26 (5.0)
Supra-levator abscess	10 (1.9)	0	0	0	0	0	0	10 (1.9)
Horseshoe abscess	41 (7.9)	0	1 (0.2)	0	0	0	3 (0.6)	45 (8.7)
Total	426 (82.4)	7 (1.4)	10 (1.9)	12 (2.3)	1 (0.2)	3 (0.6)	58 (11.2)	517 (100)

Correlation between perianal abscess types and microorganisms

**Figure 1:**
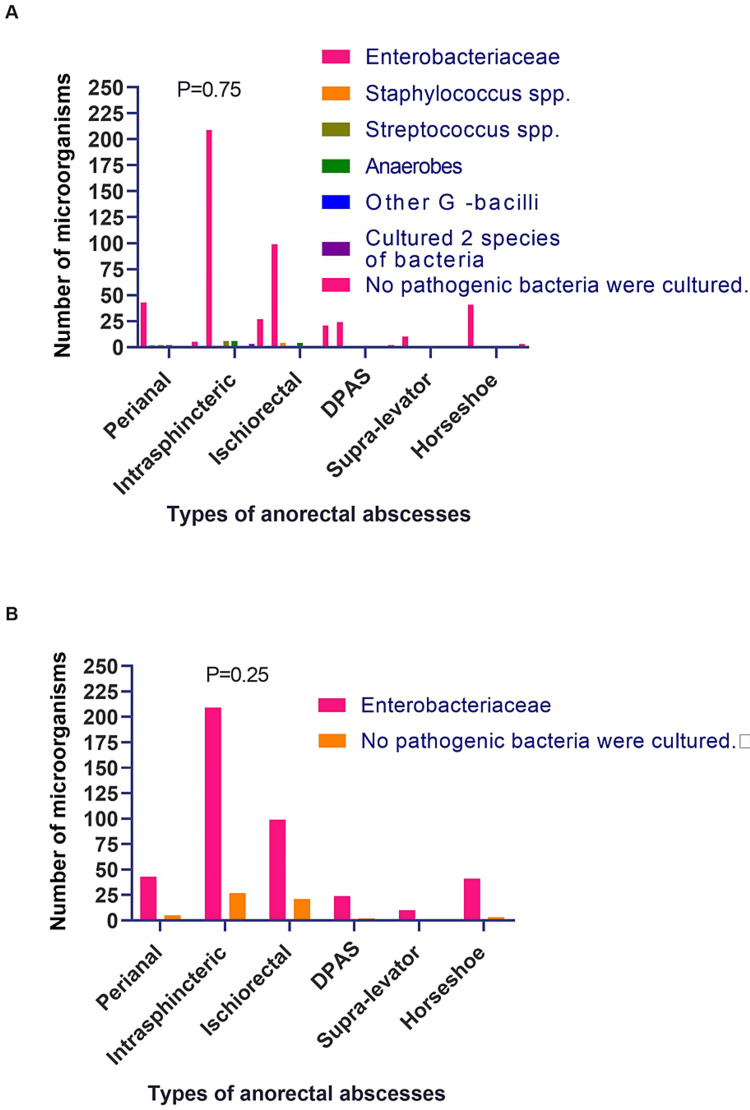
Comparison of microorganisms between different types of anorectal abscesses P<0.05 was considered to be statistically different.

As shown in Figure 1, the research team statistically analyzed the differences in the level of microbial genera between different anorectal abscess types using the chi-square test, and the statistical results showed p=0.75, which was not statistically significant (Figure 1A). The authors also compared whether there was a difference between the Enterobacteriaceae group and the No pathogenic bacteria cultured group between different anorectal abscess types, and the statistical results showed p=0.25, a statistically insignificant difference (Figure 1B). These results suggest that pathogenic microbial species and numbers do not differ significantly between different anorectal abscess types.

## Discussion

It is now widely accepted that both anorectal abscesses and anal fistulas are caused by adenogenic infections [2,7]. Since they are adenogenic infections, it is generally accepted that bacteria originating from the intestinal tract are the first to infect the anal crypts and then spread to different interstitial spaces, which in turn leads to different types of anorectal abscesses [7].

Is there some correlation between the type of anorectal abscess and the species of the causative microorganism? In other words, is there a correlation between the site of anorectal abscess and the virulence of different bacteria? With this question in mind, our research group conducted the present study. Searching the literature, the literature on pathogenic microorganisms of anorectal abscesses is very scarce with a small sample size [8-10]. Studies have focused on the use of antibiotics or not to reduce the incidence of anal fistulae after incision and drainage of anorectal abscesses. There is almost no literature to focus on the existence of a correlation between the species of causative microorganisms and the type of anorectal abscesses.

In this study, by analyzing the clinical data of pus cultures from 517 patients with anorectal abscesses, it was found that the most abundant microorganism causing the development of perianal abscesses was E. coli from the family Enterobacteriaceae, which is in agreement with previous reports [1,8]. This was closely followed by K. pneumoniae, and P. mirabilis, both of which belong to the Enterobacteriaceae family. Following them were B. fragilis, which belongs to the anaerobes, and S. aureus from the Staphylococcus spp. In this study, we also observed the data of nine patients with anorectal abscesses progressing to necrotizing fasciitis and found that the most pathogenic microorganisms were still E. coli, followed by K. pneumoniae and S. aureus, which is in agreement with the reports of previous studies [11]. These results show that the species of the causative microorganisms and their proportions are similar in both common anorectal abscesses and anorectal abscesses combined with severe complications. The incidence of necrotizing fasciitis due to anorectal infections remains high and is progressively more common in women and remains a major challenge with a high mortality rate [11]. In patients with anorectal abscesses progressing to necrotizing fasciitis, identification of the causative microorganisms by pus culture and early antibiotic treatment is highly warranted, in addition to multiple thorough debridement and drainage [11].

The use of antibiotics after incision and drainage of anorectal abscesses has been controversial. Most of the literature suggests that prophylactic antibiotic use after incision and drainage of anorectal abscesses reduces the rate of anal fistula formation [5,6,9,11,12]. Several kinds of literature support the idea of performing pus culture to identify different pathogenic microorganisms, which in turn provides rational and precise antibiotic therapy for patients with anorectal abscesses. Some literature also concluded that antibiotic therapy after drainage of anorectal abscesses is not protective against the risk of fistula formation through randomized controlled studies [13]. Consensus and guidelines state that antibiotic therapy is recommended among patients with comorbid immunodeficiency, inflammatory bowel disease, leukemia, diabetes mellitus, and some severe sepsis [2]. Recent retrospective studies have found leukopenia to be associated with an increased risk of pulmonary and hematologic complications, readmission, reoperation, discharge, and death after incision and drainage of anorectal abscesses [14]. A retrospective analysis that included 2,358 patients with incision and drainage of anorectal abscesses found common indications for readmission including recurrent/persistent abscess and fever/sepsis. Preoperative sepsis is a risk factor for reoperation and rehospitalization [15]. A study also from the United States found that inadequate antibiotic therapy leads to a higher rate of recurrence after drainage of complex perirectal abscesses and recommended providing adequate antibiotic coverage after surgical drainage [16]. A study found significant differences in the gut microbiota between healthy individuals and patients with anorectal abscesses, and the findings suggest that Klebsiella spp. is one of the potential biomarkers for diagnosing anorectal abscesses [17]. This evidence suggests that antibiotic use after incision and drainage is needed in patients with complex anorectal abscesses and anorectal abscesses with high-risk factors. In this study, we analyzed the species of the causative microorganisms and their proportions in perianal abscesses, and the results of the study may provide a basis for the rational and precise use of antibiotics in patients with the aforementioned perianal abscesses.

Also, this study found no significant difference in the species and numbers of pathogenic microorganisms between different types of anorectal abscesses. Factors affecting the type of perianal abscess are multifaceted and may include the patient's physical condition, the anatomical structure of the sphincter, and the presence or absence of early antibiotic intervention, in addition to the causative microorganisms.

The limitation of this study is that it is a retrospective study with data bias. We hope to address this issue in the future by conducting prospective studies. Also, the sample size of this study is still not large enough. We hope that future studies will include more cases and carry out validation of treatment outcomes based on microbial etiology.

## Conclusions

In conclusion, we analyzed a large sample of microorganisms from anorectal abscesses in this study. E. coli remained the most common microorganism cultured from the pus of these patients, followed by K. pneumoniae. Therefore, it is recommended to perform pus culture to identify different causative microorganisms in patients with complex anorectal abscesses, combined immunodeficiency, inflammatory bowel disease, leukemia, diabetes mellitus, necrotizing fasciitis, and sepsis present preoperatively, and to select a rational and precise antibiotic based on the species of the microorganism and its sensitivity to the drug.
